# Reparo artroscópico das lesões da rampa meniscal nas reconstruções do ligamento cruzado anterior: Nota técnica

**DOI:** 10.1055/s-0045-1809609

**Published:** 2025-06-23

**Authors:** Felipe Galvão Abreu, Sérgio Marinho de Gusmão Canuto, Vitor Barion Castro de Pádua

**Affiliations:** 1 Departamento de Ortopedia e Traumatologia, Hospital de Base, Faculdade de Medicina de São José do Rio Preto (Famerp), São José do Rio Preto, SP, Brasil; 2 Ortoclínica Hospital de Ortopedia, Maceió, AL, Brasil; 3 Departamento de Ortopedia, Faculdade de Medicina, Universidade de Marília, Marília, SP, Brasil

**Keywords:** artroscopia, joelho, menisco, sutura, arthroscopy, knee, meniscus, suture

## Abstract

Lesões meniscais são frequentemente associadas às rupturas do ligamento cruzado anterior (LCA). As lesões da rampa meniscal envolvem estruturas mais periféricas do corno posterior do menisco medial (MM) e são mais desafiadoras em relação ao seu diagnóstico e reparo em comparação com as lesões comuns do menisco. Diversas técnicas foram descritas para sutura da rampa, porém, ainda apresentando resultados insatisfatórios e com possíveis complicações. A presente nota técnica apresenta de forma clara o reparo artroscópico das lesões da rampa meniscal nas reconstruções do LCA, com auxílio de um portal posteromedial. Por proporcionar uma visão direta da lesão no momento do reparo, trata-se de uma técnica segura e com bons resultados finais.

## Abstract (en)

Meniscal injuries are frequently associated with anterior cruciate ligament (ACL) tears. Meniscal ramp injuries involve more peripheral structures of the posterior horn of the medial meniscus (MM). Their diagnosis and repair are more challenging compared with common meniscal injuries. Although several ramp suture techniques have been described, their results remain unsatisfactory, and there are potential complications. The present technical note presents the arthroscopic repair of meniscal ramp injuries in ACL reconstruction using a posteromedial portal. Since it provides a direct view of the lesion at surgery, this is a safe technique with good outcomes.

## Introdução

As lesões meniscais estão associadas à ruptura do ligamento cruzado anterior (LCA) em de 47% a 61% dos casos, sendo a região do corno posterior do menisco medial (MM) a mais frequentemente acometida. 1 2 3 As lesões da rampa meniscal consistem em fissuras periféricas longitudinais do corno posterior do MM, envolvendo suas conexões meniscocapsulares, o ligamento meniscotibial, ou ambas as estruturas. 4 Apesar de não se tratar de uma lesão recente, as lesões da rampa continuam sendo um desafio diagnóstico e terapêutico para ortopedistas e radiologistas. 5 Isso se deve, em parte, à dificuldade de identificação da lesão durante a artroscopia, e, quando identificada, de reparo da mesma. 6 Em relação ao MM, o campo visual, através exclusivamente do compartimento anterior do joelho, gera um “ponto cego,” impedindo a avaliação de até 47% da superfície do menisco, podendo, certamente, ocultar lesões mais posteriores e periféricas. Este campo visual limitado cai para 8% da superfície meniscal quando o artroscópio é inserido no compartimento posteromedial. 7

Entre as técnicas artroscópicas para reparo das lesões meniscais, o reparo *all-inside* , através de um portal anterior convencional, com implante de dispositivos de âncora de sutura meniscal, tem aumentado sua popularidade devido à sua fácil aplicação. 8 Entretanto, no caso das lesões da rampa meniscal, as técnicas de fixação de dentro para fora, de fora para dentro, ou com uso de dispositivos que utilizam âncoras, somadas à falta de visão direta sobre a lesão no momento da sutura, levam a resultados não satisfatórios, com perda de fixação, e outras complicações envolvidas com o uso das âncoras. 9 10

O objetivo do presente trabalho é descrever uma técnica de sutura das lesões da rampa meniscal, sob visão direta, através de um portal auxiliar posteromedial, assim como previamente descrito por Thaunat et al. 3 O procedimento é realizado durante as reconstruções do LCA (RLCA), nos pacientes com diagnóstico, pré- ou intraoperatório, de lesão da rampa meniscal.

## Técnica Cirúrgica

**Fig. 1:**
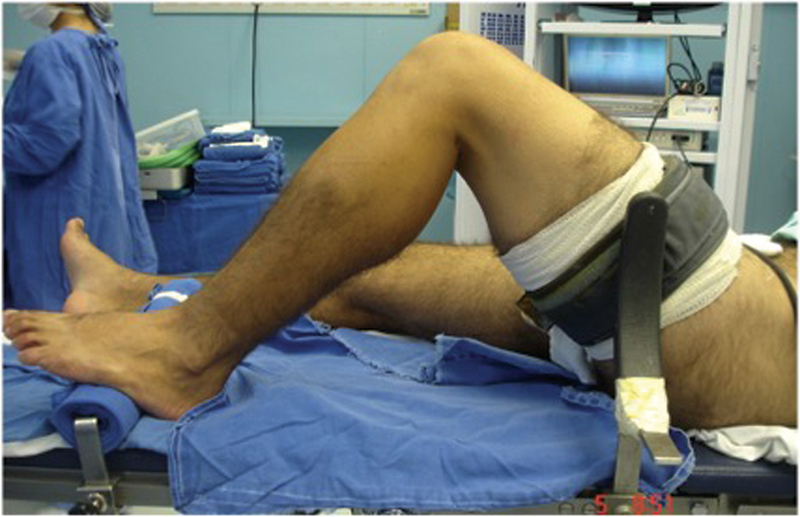
Posicionamento do membro inferior durante a reconstrução do ligamento cruzado anterior (LCA), com o pé apoiado sobre a mesa cirúrgica, apoio lateral ao nível do torniquete, e joelho com flexão de 90°.

O paciente deve ser colocado em posição supina para a artroscopia, com um apoio lateral ao nível do torniquete, e outro sob o pé, permitindo uma amplitude de movimento total, e mantendo o joelho em 90° de flexão sobre a mesa operatória, quando necessário ( Fig. 1 ). Lesões meniscais e/ou condrais são abordadas antes da reconstrução ligamentar. A decisão do tipo de enxerto específico para a RLCA é feita com base nos fatores de cada paciente e na escolha dos cirurgiões, não interferindo na abordagem do menisco.

Todo o procedimento pode ser realizado com uso de um artroscópio convencional de 30°. Inicialmente, é realizada a exploração artroscópica dos compartimentos do joelho. Nos casos em que há qualquer sinal de instabilidade meniscal, como aumento do deslocamento anterior do mesmo sob tração, ou quando identifica-se a presença de alguma fissura em seu folheto inferior, a lesão da rampa deve ser suspeitada. Realizamos de rotina a exploração sistemática do compartimento posteromedial em todas as cirurgias de RLCA, como descrito por Sonnery-Cottet et al., 11 que consiste em três passos: (1) a exploração pelos portais anteriores, com um probe testando a estabilidade do menisco medial; (2) a exploração visual do compartimento posteromedial; e (3) a criação do portal posteromedial, para investigar uma possível lesão com auxílio de uma agulha ou probe.

**Fig. 2:**
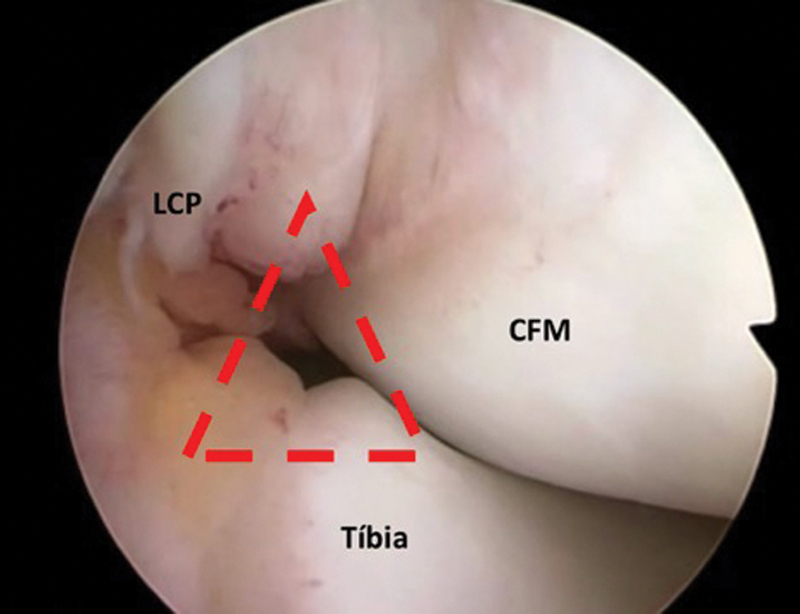
Imagem artroscópica identificando o espaço no intercôndilo por onde é inserido o artroscópio para acesso ao compartimento posteromedial do joelho. O ponto correto é identificado no centro de um triângulo (em vermelho) formado pelo côndilo femoral medial (CFM), ligamento cruzado posterior (LCP) e tíbia.

**Fig. 3:**
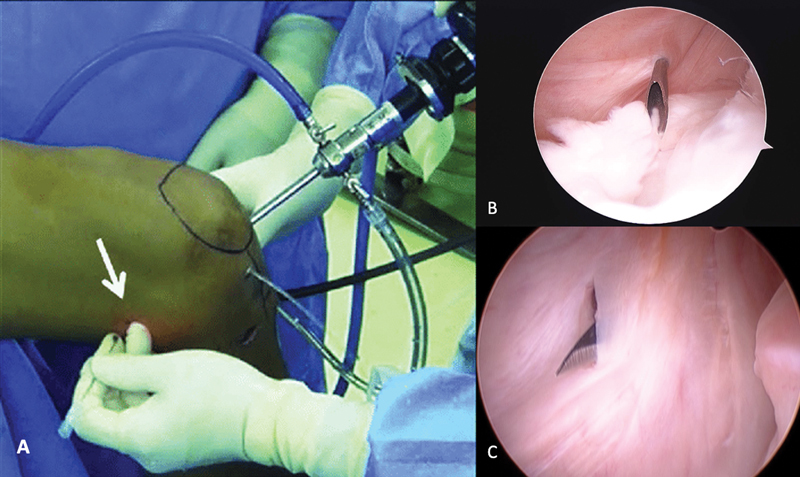
Detalhes da confecção do portal posteromedial. O uso da transiluminação evita a lesão iatrogênica de vasos e nervos ( **A** ). A agulha é introduzida na direção da lesão, para a definição do melhor ponto para criar o portal ( **B** ). Sob visão direta, o portal é criado com uso de uma lâmina de bisturi ( **C** ).

**Fig. 4:**
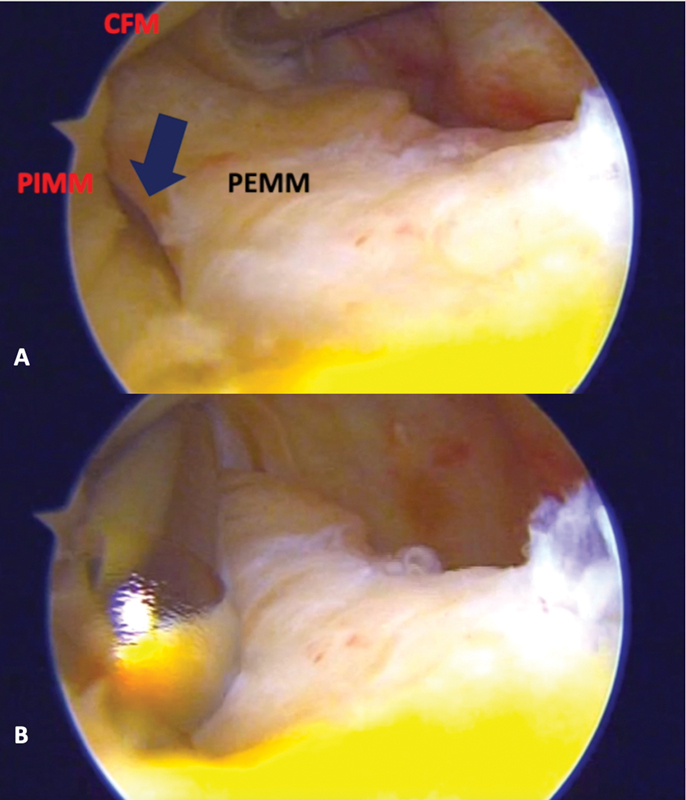
Imagem artroscópica ( **A** ) mostrando uma clara visualização da lesão da rampa meniscal (seta preta), da porção externa do menisco medial (PEMM), da porção interna do menisco medial (PIMM) e do CFM. A cruentização e regularização das bordas da lesão da rampa meniscal são realizadas com uma lâmina de shaver ( **B** ).

Para a avaliação do compartimento posteromedial do joelho, o artroscópio é mantido no portal anterolateral, e inserido através de um espaço no intercôndilo, definido pelo côndilo femoral medial, ligamento cruzado posterior (LCP) e tíbia ( Fig. 2 ). A realização de uma manobra em valgo pode facilitar a passagem do artroscópio. A confecção do portal posteromedial é feita com o joelho em 90° de flexão. O uso da transiluminação auxilia na visualização de veias a nervos que devem ser preservados. Uma agulha é introduzida na direção da lesão, logo acima dos tendões flexores e 1 cm posterior à linha articular femorotibial medial. Em seguida, a incisão é realizada com um bisturi de lâmina n° 11 sob visão direta artroscópica ( Fig. 3 ). Neste ponto, a rotação interna do pé é realizada, gerando uma posteriorização do côndilo tibial medial, e expondo, assim, a lesão com maior facilidade. A lâmina de shaver é, então, inserida através do portal posteromedial e ambas as superfícies da lesão devem ser cruentizadas ( Fig. 4 ).

**Fig. 5:**
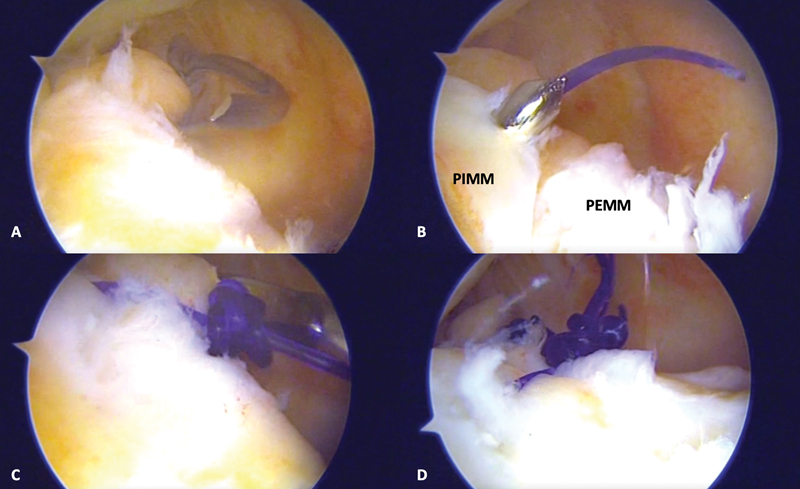
O gancho de sutura de 25° (SutureLasso, Arthrex) é introduzido pelo portal posteromedial para fazer o reparo da lesão ( **A** ). Após atravessar a PEMM e a PIMM, o fio é liberado pelo gancho de sutura ( **B** ). A sutura é feita com uso de pontos simples, e com auxílio de um empurrador de nós ( **C** ). Aspecto final da lesão da rampa após realização do reparo ( **D** ).

Para a realização das suturas utiliza-se um gancho de sutura de 25° (SutureLasso, Arthrex, Naples, FL, Estados Unidos) para a esquerda nos joelhos direito, e para a direita nos joelhos esquerdo, carregado com um fio de monofilamento absorvível n° 1 (PDS; Ethicon, Inc., Raritan, NJ, Estados Unidos). O gancho de sutura é manipulado pelo cirurgião de maneira que a ponta afiada penetre no fragmento mais periférico da lesão, com a parte capsular, em toda sua espessura. Em seguida, o gancho de sutura é passado da mesma forma, através da parte central do menisco medial. Nesse ponto, a técnica aqui descrita assemelha-se a um reparo artroscópico do ombro com uma lesão de Bankart. 12 O fio é liberado pelo gancho, e sua extremidade livre é, então, alcançada com uma pinça artroscópica e recolhida pelo portal posteromedial. A confecção dos pontos é realizada utilizando qualquer tipo de nó deslizante (conforme a preferência do cirurgião) e com o auxílio de um empurrador de nós ( Fig. 5 ). De modo geral, um a três pontos são suficientes para o reparo completo da lesão, deixando uma distância de, aproximadamente, 1 cm entre eles. O reparo satisfatório e estável deve ser confirmado por avaliação com uso do probe, inserido e visualizado pelos portais anteriores e posteromedial. Por fim, o procedimento de RLCA pode ser realizado conforme a técnica escolhida pelo cirurgião.

## Reabilitação

A extensão total do joelho e a ativação do quadríceps são elementos chave na fisioterapia precoce. Os pacientes submetidos ao reparo da lesão da rampa devem ser orientados a deambular com carga parcial, e auxílio de muletas, por 6 semanas. A amplitude de movimento também é limitada a 90° nas primeiras 6 semanas, com aumento progressivo a partir de então. A bicicleta ergométrica é permitida após 2 meses. Esportes que não demandam mudanças de direção (ex.: corrida, ciclismo) podem ser liberados em 4 meses, enquanto aqueles que demandam (ex.: dança, vôlei) em 6 meses. Os esportes de contato (ex.: futebol, handebol e lutas) são liberados entre 8 e 9 meses. 3

## Comentários Finais

A taxa de falha no reparo das rupturas do corno posterior do MM, incluindo as lesões da rampa, permanece alta, apesar do desenvolvimento de dispositivos de sutura *all-inside* . 8 A técnica aqui descrita busca eliminar algumas possíveis causas dessas falhas, trazendo algumas vantagens em relação ao uso dos dispositivos. A exploração do menisco medial sob visão direta, no compartimento posteromedial, não só melhora consideravelmente o diagnóstico da lesão da rampa, como também proporciona uma excelente visão da mesma durante o reparo, possibilitando um melhor desbridamento das bordas e um melhor controle do fechamento completo da lesão. O que vemos na prática é que, em diversos casos, durante a exploração do compartimento posteromedial, encontramos a borda mais externa da lesão caída atrás do planalto tibial. Nesses casos, não se faz possível qualquer tipo de reparo realizado pelo compartimento anterior do joelho, visto que o dispositivo utilizado vai atravessar a porção interna da lesão, e se fixar na cápsula articular, um tecido friável e pouco resistente, deixando a lesão não reparada. Sem a visão direta da lesão, entendemos que as tentativas de reparo dessas lesões são feitas às cegas. A melhor visualização também permite a colocação de suturas verticais perpendiculares às fibras profundas dos meniscos, envolvendo o ligamento meniscotibial, e gerando uma melhor estabilidade do pós-reparo. A redução da lesão é visualizada durante o procedimento, o que não é possível no implante *all-inside* . Ainda, apesar do menor custo no uso do gancho de sutura 25°, o mesmo dispositivo pode ser usado para realizar mais de uma sutura, diferentemente dos dispositivos com âncoras.

Como toda técnica cirúrgica, esta não está isenta de desvantagens ou riscos. Por exigir uma técnica mais apurada do cirurgião, entendemos que existe uma curva de aprendizado significativa para tal. Isso reforça a importância de sistematizar uma rotina de exploração do compartimento posteromedial durante as RLCA, o que fará com que o cirurgião se familiarize com o artroscópio inserido no compartimento posterior do joelho, e melhore sua sensibilidade na identificação dessas lesões, muitas vezes ainda subdiagnosticadas nos exames de imagem. A presença de uma incisão adicional, com a criação do portal posteromedial é também uma desvantagem da técnica. Apesar do risco de lesão do nervo e veia safena, o uso da transiluminação, durante a confecção do portal auxilia e evita a lesão destas estruturas.
